# Oxford Cognitive Screen – Brazilian Portuguese version (OCS-Br) A pilot study

**DOI:** 10.1590/1980-57642018dn12-040014

**Published:** 2018

**Authors:** Claudia Cristina Ferreira Ramos, Daniel Krempel Amado, Conrado Regis Borges, Eduardo Bergamaschi, Ricardo Nitrini, Sonia Maria Dozzi Brucki

**Affiliations:** 1 Cognitive and Behavioral Neurology Group – Hospital das Clínicas, Universidade de São Paulo, São Paulo, SP, Brazil.

**Keywords:** stroke, cognitive screening, normative data, Oxford Cognitive Screen, AVC, avaliação cognitiva, dados normativos, Oxford Cognitive Scale

## Abstract

Cognitive impairment is very common in stroke patients and underdiagnosed. Symptoms such as language, praxis, visuospatial, visuoconstructive and memory impairment are prominent. The screening cognitive tests available do not address some specific characteristics of stroke patients and have major limitations in relation to the most impaired cognitive domains.

**Objective:**

To test the applicability of a Brazilian version of the Oxford Cognitive Screen in a convenience sample of individuals with normal cognition.

**Methods:**

Thirty neurologically healthy participants underwent the OCS-Br in this pilot study.

**Results:**

The mean score on each task was: naming: 3.4 (SD=0.72) (maximum value of 4); semantics: 3 (SD=0) (maximum 3); orientation: 4 (SD=0) (maximum 4); visual field: 4 (SD=0) (maximum 4); sentence reading: 14.53 (SD: 1) (maximum 15); number writing: 2.86 (0.6) (maximum 3); calculation: 3.8 (SD=0.48) (maximum 4); and accuracy on the broken hearts test: 47.3 (3.3) (maximum 50). The scores obtained were similar to those of the English original sample.

**Conclusion:**

We observed similar values on each separate OCS task in comparison to the original test, confirming that the Brazilian Portuguese version is comparable to other studies.

## RESUMO

O comprometimento cognitivo é muito comum em pacientes com AVC e pouco diagnosticado. Déficits cognitivos envolvendo as funções de linguagem, praxia, habilidades visuoespaciais, visuoconstrutivas e memória são proeminentes. Os testes de avaliação cognitiva disponíveis não abordam algumas características específicas dos pacientes com AVC e apresentam limitação importante em relação aos domínios cognitivos mais comprometidos.

**Objetivo:**

Testar a aplicabilidade de uma versão brasileira do Oxford Cognitive Screen em uma amostra de conveniência com indivíduos sem prejuízo cognitivo.

**Métodos:**

Trinta participantes neurologicamente saudáveis foram submetidos ao OCS-Br neste estudo piloto.

**Resultados:**

O escore médio em cada tarefa foi: nomeação: 3,4 (DP=0,72) (valor máximo de 4); semântica: 3 (DP=0) (máximo de 3); orientação: 4 (DP=0) (máximo de 4); campo visual: 4 (DP=0) (máximo de 4); leitura de sentenças: 14,53 (DP: 1) (máximo de 15); número escrito: 2,86 (0,6) (máximo de 3); cálculo: 3,8 (DP=0,48) (máximo de 4); precisão no teste de corações partidos: 47,3 (3,3) (máximo de 50). Os escores foram semelhantes aos da amostra original.

**Conclusão:**

Observamos valores similares em cada tarefa separada do OCS em comparação ao teste original, o que torna a versão em português brasileiro comparável a outros estudos, e todos os itens foram bem compreendidos com potencial de usabilidade em nosso país.

Strokes are among the leading causes of death and disability worldwide, representing the 4^th^ most common cause of mortality in Brazil.^1^

About 48% of stroke survivors present cognitive deficits in the early phase following a stroke and have an increased risk of developing dementia.^2^^,^ 3 Cerebral small vessel disease frequently causes cognitive impairment (in 36-67% of cases), as does lacunar stroke (in approximately half of cases).^4^

Cognitive deficits include language, spatial attention, memory, praxis, executive functions and speed of information processing as the most frequent sequelae.^5^ Stroke guidelines recommend identifying cognitive deficits soon after stroke onset to allow planning of the most appropriate rehabilitation program, since cognitive impairment predicts poor prognosis in the long term.^6^

Vascular Dementia is the second most common cause of dementia, having a prevalence ranging from 8.7% to 26.5% in Latin America.^7^^,^ 8 In a cross-sectional Brazilian clinicopathological study, forty-four percent of 1,092 participants received a neuropathological diagnosis of dementia, of which 35% were Vascular Dementia.^9^ Epidemiologic studies in Brazil reveal prevalences of between 9.3% and 15.9% for vascular dementia among demented participants,^10^ whereas studies in tertiary outpatient clinics report a prevalence of vascular dementia of between 24.9 and 32.25%, and of 36.9% in a sample with presenile dementia.^11^^-^ 13

Vascular cognitive impairment (VCI) was observed in 16.8% of non-embolic ischemic stroke patients during a follow-up of 12 months (4.6% were diagnosed as cognitive impairment no dementia and 12.2% as dementia).^14^ However, there seems to be no gold standard for cognitive screening in this population.

The Mini-Mental State Examination (MMSE) and the Montreal Cognitive Assessment (MoCA) are widely used to assess cognition post-stroke although these instruments have low sensitivity for evaluating the most commonly affected cognitive domains after stroke, such as executive function and processing speed.^15^^-^ 18 Moreover, poststroke cognitive impairment is usually accompanied by additional cognitive deficits, such as aphasia/language dysfunction, vision loss, apraxia and spatial neglect, for which the above-mentioned tests are not sensitive and may greatly underestimate cases of cognitive impairment involving specific neurological deficits (e.g.: aphasia).

Considering this context, Demeyere et al. developed the Oxford Cognitive Screen Test (OCS), a stroke-specific short cognitive screening tool, which evaluates the main cognitive domains impaired in these patients.^19^ The test has been validated in Hong-Kong, Italy and South Africa.^20^^-^ 22 The OCS allows detailed screening of the cognitive deficits after stroke, through the separate assessment of number cognition, praxis, executive functions, memory, language and attention.

The aim of this study is to test a Brazilian population without known cognitive impairment using the OCS-Br.

## METHODS

Thirty neurologically healthy participants underwent the OCS-Br in this pilot study. Participants were enrolled from August to November, 2017 at the Hospital das Clinicas, Universidade de São Paulo. All participants were native Portuguese speakers and screened for cognitive impairment using the Mini-Mental State Examination scores, according to normative values, adjusted for education available for the Brazilian population^23^ and for preserved activities of daily living, using Pfeffer’s Functional Activities Questionnaire.

Only individuals without any reported history of neurological disease, speech and language problems, reading impairment, and/or learning difficulties were included in the current study.

The screening for depression was defined as positive when frequent sadness or anhedonia were present. History of neurological and psychiatric conditions, as well as the use of psychotropic drugs, was also assessed. All patients signed an informed consent form.

### The Oxford Cognitive Screen

This tool consists of ten sub-tests encompassing various cognitive domains: attention and executive function, memory, language, praxis, number processing, and visuospatial function. The scale is designed to be completed with only one hand in order to reduce the impact of upper limb motor weakness.

The OSC scale can be applied within 15 minutes, performed at bedside and used in relatively acute phases of a stroke (after 3 days from onset).

The OCS-Br, a version of OCS, was first translated into Brazilian Portuguese by three independent Portuguese-English proficient speakers. Minimal cultural modifications of selected test items were then made and a back translation performed by a native English speaker.

All items were translated following specific rules dictated by the original authors, who agreed to its use for the Brazilian process of validation.

## RESULTS

We evaluated 30 neurologically healthy participants (Table 1). Although six individuals had a previous diagnosis of psychiatric illness (two with depression, two with anxiety and one with panic disorder), they were considered asymptomatic at the time of evaluation. Three of these patients were still receiving pharmacological treatment. The diagnosis was reached using the DSM-IV criteria.

**Table 1:** Demographic data.

N=30	Mean	SD	Range
Sex (female)	20 (66.7%)		
Age	61.19	6.31	46-72
Education (years)	13.67	4.87	4-22
MMSE	28.26	1.85	23-30

The mean score for each task is depicted in Table 2.

**Table 2:** Scores on subitems of OCS-Br compared to the original version.

Task name	Measure	OCS-Br Mean score (SD)	Original OCS (Demeyere, 2015)	Maximum score
Picture naming	Overall accuracy	3.4 (0.72)	3.82	4
Semantics	Overall accuracy	3 (0)	3	3
Orientation	Overall accuracy	4 (0)	4	4
Visual fields	Overall accuracy	4 (0)	4	4
Sentence Reading	Overall accuracy	14.53 (1)	14.85	15
Number writing	Overall accuracy	2.86 (0.6)	2.93	3
Calculation	Overall accuracy	3.8 (0.48)	3.9	4
Broken hearts	Overall accuracy	47.3 (3.3)	47.31	50
Imitation	Overall accuracy	11.1 (1.4)	10.84	12
Recall and recognition	Verbal recall	2.83 (0.9)	2.52	4
	Verbal memory recall/recognition	3.89 (0.3)	3.72	4
	Episodic recognition	3.86 (0.3)	3.83	4
Executive task	Mixed score (trails)	10.4 (3)	10.4	12
	Executive score (trails)	1.3 (3)	1.36	---

## DISCUSSION

Vascular cognitive impairment is characterized by a heterogeneous group of subtypes relating clinical and neuroimaging aspects, which may be associated with a group of “focal”, circumscribed impairment of specialized functions such as language (aphasia), intentional movements (apraxia), or categorical recognition (agnosia), among others. A brief test, and oftentimes a bedside test, are necessary to evaluate these patients. Thus, standardized screening tools are important to evaluate stroke survivors and predict functional outcome, thereby enabling suitable rehabilitation programs^24^. The OSC scale was created in order to measure commonly occurring problems after stroke and minimize confounding with aphasia and neglect. A major advantage of the OCS is its capacity to evaluate aphasic and/or hemiplegic patients, while the scale also incorporates assessment of apraxia and neglect^25^.

In this pilot study, we observed similar values on each separate OCS task compared to the original test, confirming the comparability of the Brazilian Portuguese version.

The OCS is a short version of the Birmingham Cognitive Screen (BCoS) which contains twenty-three subtests and examines five cognitive domains, including language, memory, praxis, calculus, and executive functioning and attention. The scale takes about 45 to 75 minutes to perform^26^ and also evaluates the cognitive domains of reading, writing, praxis and spatial neglect.

Kong et al., validated the Hong Kong version of the Oxford Cognitive Screen (HK-OCS), whose control group had a similar performance to our version.^20^ The participants were assessed using the Hong Kong version of the following assessments: Western Aphasia Battery, MMSE, MoCA, Modified Barthel Index, and Lawton Instrumental Activities of Daily Living scale. Most subtests in the HK-OCS correlated significantly with subtests assessing similar cognitive domains of other assessment tools, with the exception of Verbal Memory (recall and recognition), Episodic Memory, and the Hearts test of neglect. Specific HK-OCS subtests, including semantics, episodic memory, number writing, and orientation, were the best predictors of functional outcomes.

Humphreys GW et al.^22^ reported a new version of the OCS designed for low-literacy settings. The OCS-Plus, a tablet-based approach to cognition assessment, was validated in a population of older adults in rural South Africa and analyzed in relation to other factors including physical and mental health, age, education, and alcohol consumption. Results indicated that the OCS-Plus shows high task compliance and good validity, improving the measurement of cognition with minimal language content, thereby avoiding floor and ceiling effects present in other short cognitive screens.

It is also important to highlight the shortcomings of the use of tests originally designed for dementia in populations with stroke. Studies demonstrate greater sensitivity of the OCS scale over the MMSE scale, with a much higher incidence of cognitive impairment detected using the OCS (91% at least one domain impairment) than the MMSE (35%).^23^ There is also evidence of better performance of these patients on the evaluation of sub-items of the OCS compared to the MoCA^27^ and there is also evidence of greater sensitivity of the MoCA over the MMSE.^14^ Overall, it appears that cognitive deficits following stroke are more effectively detected by specially designed tools to capture deficits following stroke than by tools originally designed for dementia.^28^^,^ 29

The population tested in this pilot study was relatively highly educated (mean 13.67 years) and thus does not reflect the Brazilian reality. However, further studies involving a greater number of patients, wider ranges of age and education and inclusion of patients in the setting of acute and chronic ischemic brain disease will be needed to validate this test in the Brazilian population.
